# Inverted Meckel’s diverticulum: a case report 

**DOI:** 10.1186/s13256-021-02736-2

**Published:** 2021-05-22

**Authors:** Lovenish Bains, Rahul Bhatia, Rohit Kaushik, Pawan Lal, Gayatri Rajpaul, Veerpal

**Affiliations:** Department of General Surgery, Maulana Azad Medical College, New Delhi, India

**Keywords:** Inverted Meckel’s diverticulum, Intussusception, Lipoma, Obstruction

## Abstract

**Background:**

Inverted Meckel’s diverticulum refers to the condition in which the diverticulum inverts on itself. The reasons for such an inversion are poorly understood due to the rarity of the condition. We present a case of inverted Meckel’s diverticulum, an uncommon finding, as a cause of recurrent intussusception.

**Case presentation:**

A 30-year old Indian woman presented with complaints of pain in the central abdomen for 3 days, accompanied with vomiting and loose stools. Computed tomography images were suggestive of intussusception with intestinal obstruction. Intra-operative findings were suggestive of an intussuscepted segment of ileum measuring 10 cm in length, proximal to ileocecal junction. Ileo–ileal anastomosis was performed after appropriate resection. Upon opening the specimen, we were surprised to find an inverted Meckel diverticulum with lipoma at one end causing the intussusception. The patient made an uneventful recovery and was discharged after 5 days.

**Conclusion:**

The reasons for inversion include abnormal peristalsis around the diverticulum and non-fixity of the diverticulum itself. The inverted diverticulum itself can cause luminal compromise and acts as a lead point for intussusception leading to obstruction. Computed tomography remains the diagnostic tool of choice for identifying intestinal obstruction and intussusception. Although pathological signs, such as lipoma, can be identified, the identification of any inversion will require a proficient radiologist. Inverted Meckel’s diverticulum is a rare condition which is difficult to diagnose preoperatively. Treatment is surgical, whether diagnosed pre-operatively or intra-operatively, and includes segmental resection and anastomosis. This uncommon condition should be noted as one-off differential diagnosis for intussusception and intestinal obstruction.

## Introduction

Meckel’s diverticulum, although a common anomaly of the gastrointestinal tract, rarely presents with symptoms. Symptoms, when present, signify an underlying disease process, suggesting complications of Meckel’s diverticulum, such as intestinal obstruction, intussusception, bleeding, and inflammation [1, 2]. An inverted Meckel’s diverticulum refers to the condition in which the diverticulum inverts on itself. Very few cases of inverted Meckel’s diverticulum have been reported in the indexed literature to date, and it is an uncommon condition that is rarely encountered in medical practice [3]. This novel entity is clinically challenging to diagnose pre-operatively. We present our experience with a case of recurrent intestinal obstruction due to an inverted Meckel’s diverticulum with a lipoma at one end, and discuss factors responsible for this condition.

## Case presentation

**Fig. 1:**
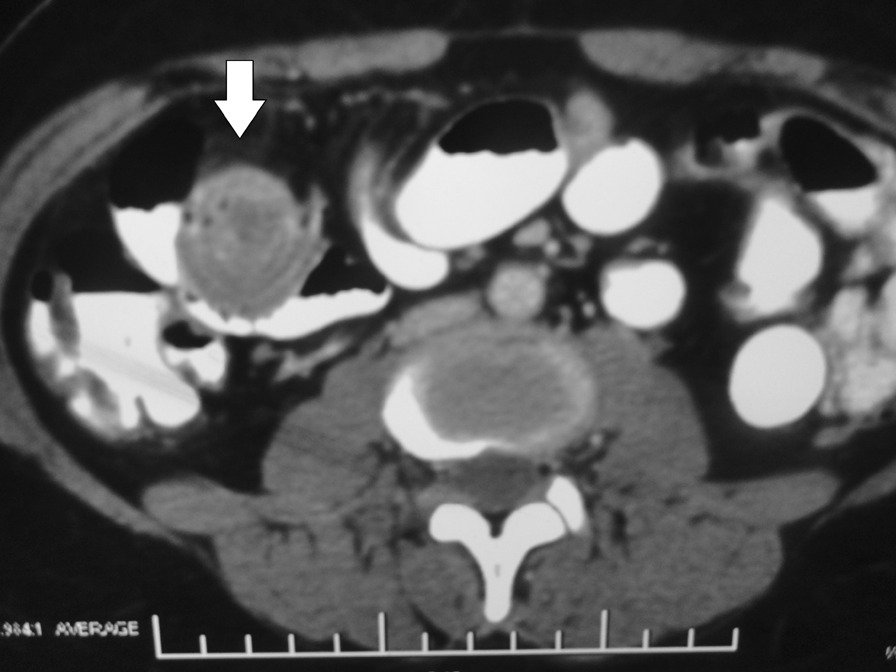
Computed tomography image (transverse plane) showing whorled pattern (arrow) suggestive of intussusception

**Fig. 2:**
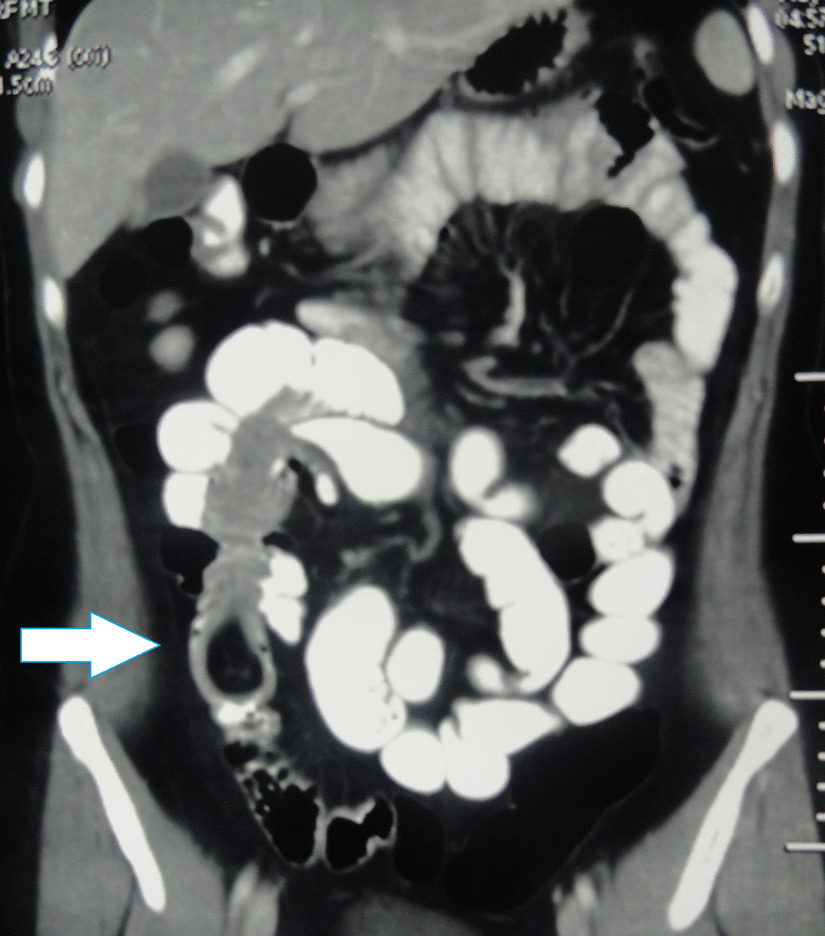
Coronal section showing hypodense intraluminal content suggestive of lipoma (arrow)

**Fig. 3:**
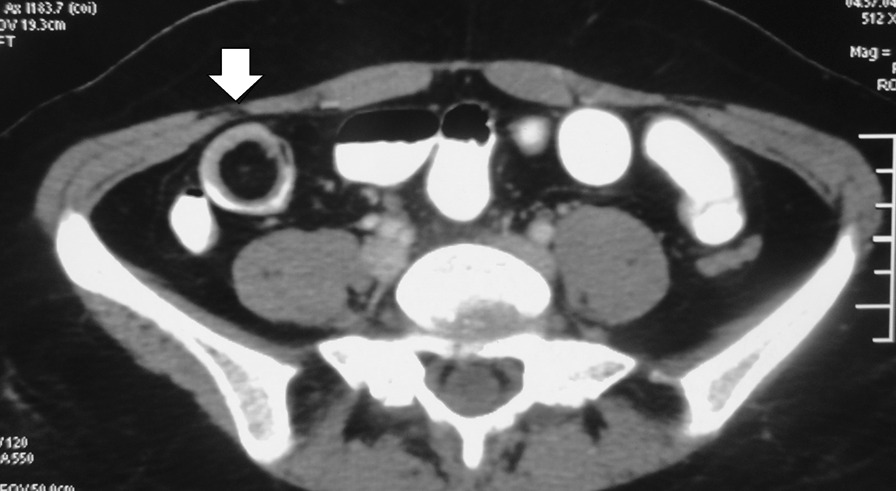
Computed tomography image (transverse plane) showing classical target sign (arrow)

**Fig. 4:**
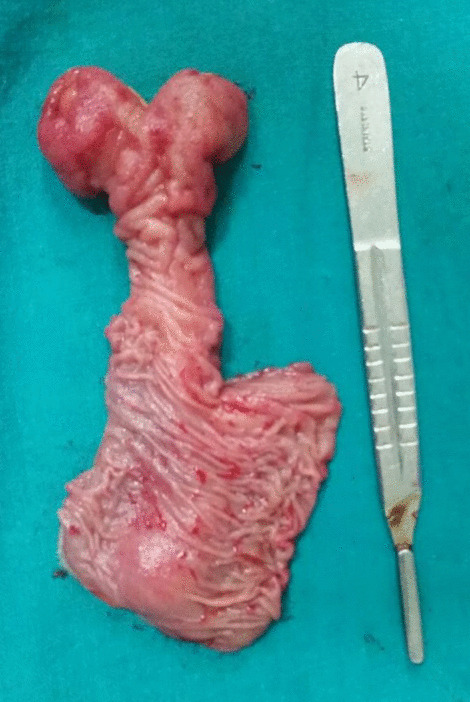
Resected segment showing inverted diverticulum with bulbous swelling at the end

**Fig. 5:**
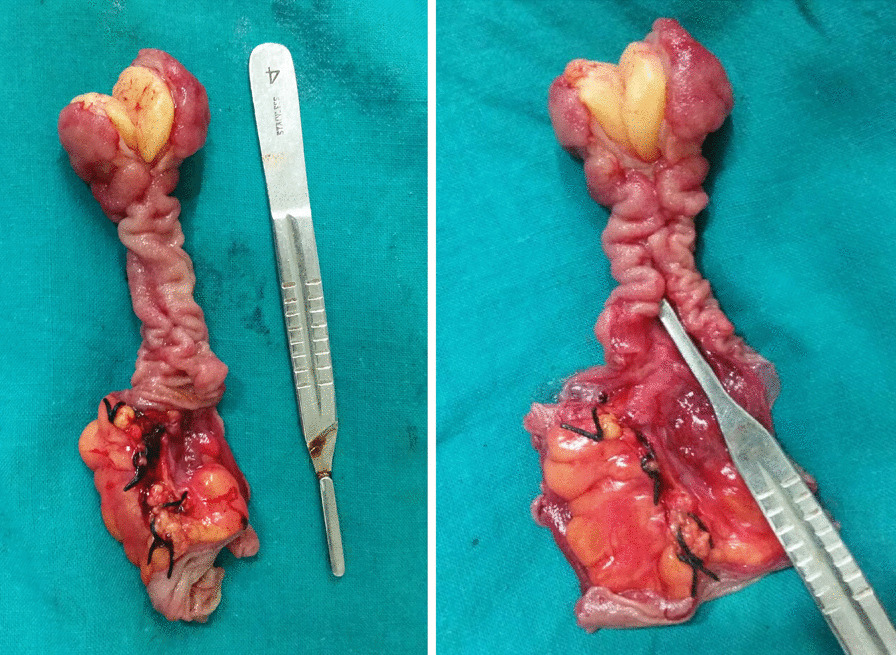
Inverted Meckel’s diverticulum with lipoma at its end

A 30-year old Indian woman presented with complaints of pain in the central abdomen for 3 days, with vomiting and loose stools. On examination, she had a pulse rate of 84/min, blood pressure of 110/84 mmHg, respiration rate of 15 breaths per minute and SpO_2_ of 98% at room air. The abdomen was soft, there was mild central distension, and bowel sounds were increased. Laboratory investigations were essentially normal. The patient had a history of two similar episodes over the preceding 6 months, both of which resolved spontaneously following treatment with a conservative regimen. Abdomen X-ray was suggestive of dilated jejunal and proximal ileal loops. In view of the previous two similar episodes, a computed tomography (CT) was done, with the results suggestive of an intraluminal fatty mass surrounded by a thick collar of soft tissue attenuation consistent with the target sign suggesting intussusception in the distal small bowel, along with dilated proximal small bowel loops. (Figs. 1, 2, 3) The patient underwent laparoscopic procedure. Intra-operative findings were suggestive of an intussuscepted segment of ileum measuring 10 cm in length, about 75 cm proximal to the ileo-cecal junction, with dilated proximal ileal and jejunal loops. The segment was removed through a small midline incision at the umbilicus. A firm mass was palpable within the lumen; hence, an enterotomy was performed, revealing a tubular segment of 4 cm in length with a globular swelling at the end. The involved segment was resected, and an ileo-ileal anastomosis performed, then reposited back to the peritoneal cavity and the port sites closed. Further careful examination of the specimen revealed that it was a narrow-mouth, inverted diverticulum with lipoma at its end, leading to intussusception and obstruction and causing the recurrent episodes (Figs. 4, 5). Histopathology investigations revealed a necrotic and hemorrhagic architecture of the resected bowel segment that was consistent with intussusception, along with features of normal small bowel mucosa within the diverticulum, and a lipoma within. The patient made an uneventful recovery and was discharged after 5 days. At the 5-month follow-up, the patient was in good health.

## Discussion

Meckel’s diverticulum is a remnant of the vitelline duct [3], and it is the most frequently encountered anomaly, with a reported incidence of 1–3% in the general population. The most common presenting complaints, in order of decreasing frequency, are bleeding, anemia, and abdominal pain [1, 3, 4], associated with complications of bleeding, intestinal obstruction, intussusception and rarely hernia or perforation [5]. Intestinal obstruction as a consequence of Meckel’s diverticulum accounts for 20–25% of presenting cases [4].

An inverted Meckel’s diverticulum refers to the condition in which the diverticulum inverts on itself. The plausible causal factor of this inversion is an abnormal peristalsis in the segment of bowel just proximal to the diverticulum [6]. In addition, the inverted diverticulum itself acts as a lead point for intussusception in such patients, thereby posing as an impediment to bowel function, leading to obstruction [6, 7]. Another explanation is that because Meckel’s diverticulum is not fixed to the mesentery or the intestine, it increases the likelihood of an inversion [3]. Furthermore, the presence of lipoma within inverted Meckel’s diverticulum increases the chance of intussusception and luminal compromise depending upon the size of the mass. To our knowledge, there have been fewer than 50 reports of inversion of Meckel’s diverticulum in the indexed literature to date, making it a rare finding, with the number of cases that were diagnosed pre-operatively representing a miniscule proportion of the total reported [3].

This condition may present with pain, bleeding, luminal compromise, and/or intussusception. The CT scan of a patient with Meckel’s diverticulum may show an air- or fluid-filled blind ending pouch from the anti-mesenteric surface of the distal ileum, whereas that of an inverted Meckel’s diverticulum appears as an intraluminal mass surrounded by a thick collar of enhancing soft tissue due to the entrapped perienteric fatty tissue within the inverted serosal side of the diverticulum [7]. A CT scan not only helps the treating physician(s) to confirm the diagnosis, but also helps to determine the condition of the bowel wall in intussusception along with any other concomitant pathology [8]. Capsule endoscopy can also facilitate the diagnosis, but is associated with limitations in identifying the exact location of the intestinal pathology and increased risk of intestinal obstruction [9]. Double balloon enteroscopy is also being increasingly used for the diagnosis as it enables biopsy of the intestine but it is a technically demanding methodology [10, 11]. Other modalities, including small bowel barium series and ultrasonography, have proven to be beneficial but these have been replaced by the CT scan as the first investigation of choice as CT can pick up intra- and extra-luminal pathology upon examination of the whole abdomen.

Our patient presented with complaints of central abdominal pain and features of bowel obstruction for the third time in the last 6 months. CT imaging showed features of an intraluminal mass with a thick collar of surrounding soft tissue density and target sign on the CT scan, corresponding well with the described findings. The suspected mass within was identified as lipoma due to its classic homogenous low attenuation appearance on the CT scan with a minimal internal soft tissue component [12]. However, entrapped perienteric fatty tissue may give a pseudo-lipoma appearance [13–16]. Lipomas of the small intestine are the second most common benign tumors after leiomyomas, and 50% are found in the ileum. They appear as a sessile mass or polyp from the submucosal layer towards the intestinal lumen [17], and they may extrude into the bowel wall or through the luminal area due to motor activity of the underlying muscularis propria, causing inversion of the Meckel’s diverticulum, as in our case. Intussusception caused by inverted Meckel’s diverticulum containing a lipoma within is a very rare condition, with only a few cases reported in the indexed literature, accounting for its rarity [5, 7].

Surgery remains the mainstay of treatment of adult intussusception due to any cause. The surgical procedure of choice remains segmental resection followed by primary anastomosis [8]. In our case, we were able to localize the lesion laparoscopically followed by extracorporeal resection and anastomosis, thus minimizing the morbidity associated with a large laparotomy incision, causing lesser post-operative pain, and enabling early recovery.

## Conclusion

Inverted Meckel’s diverticulum is an uncommon condition which results due to abnormal peristalsis around the diverticulum and non-fixity of the diverticulum itself. The aim of this case report is to alert surgeons to this rare clinical entity which is encountered only on the surgical table. Pre-operative diagnosis of inverted Meckel’s diverticulum is a difficult due to overlapping clinical and imaging features. Treatment is surgical, whether diagnosed pre-operatively or intra-operatively, and includes segmental resection and anastomosis.

## Data Availability

Not available.
